# Right Lateralized Brain Reserve Offsets Age-Related Deficits in Ignoring Distraction

**DOI:** 10.1093/texcom/tgaa049

**Published:** 2020-08-20

**Authors:** Nir Shalev, Méadhbh B Brosnan, Magdalena Chechlacz

**Affiliations:** Department of Experimental Psychology, University of Oxford, Oxford OX2 6GG, UK; Oxford Centre for Human Brain Activity, University of Oxford, Oxford OX3 7JX, UK; Wellcome Centre for Integrative Neuroimaging, University of Oxford, Oxford OX3 7JX, UK; Department of Experimental Psychology, University of Oxford, Oxford OX2 6GG, UK; Oxford Centre for Human Brain Activity, University of Oxford, Oxford OX3 7JX, UK; Wellcome Centre for Integrative Neuroimaging, University of Oxford, Oxford OX3 7JX, UK; Turner Institute for Brain and Mental Health and the School of Psychological Sciences, Monash University, Melbourne, Australia; Centre for Human Brain Health, University of Birmingham, Birmingham B15 2TT, UK; School of Psychology, University of Birmingham, Birmingham B15 2TT, UK

**Keywords:** aging, cognitive reserve, education, saliency, visual attention

## Abstract

Age-related deterioration of attention decreases the ability to stay focused on the task at hand due to less efficient selection of relevant information and increased distractibility in the face of irrelevant, but salient stimuli. While older (compared with younger) adults may have difficulty suppressing salient distractors, the extent of these challenges differs vastly across individuals. Cognitive reserve measured by proxies of cognitively enriching life experiences, such as education, occupation, and leisure activities, is thought to mitigate the effects of the aging process and account for variability in trajectories of cognitive decline. Based on combined behavioral and neuroimaging (voxel-based morphometry) analyses of demographic, cognitive, and neural markers of aging and cognitive reserve proxy measures, we examine here predictors of variability in the age-related changes in attention function, indexed by ability to suppress salient distraction. Our findings indicate that in healthy (neurotypical), aging gray matter volume within several right lateralized fronto-parietal brain regions varies according to both levels of cognitive reserve (education) and the capacity to effectively select visual stimuli amid salient distraction. Thus, we provide here novel experimental evidence supporting Robertson’s theory of a right lateralized neural basis for cognitive reserve.

## Introduction

Cognitive decline is frequently associated with aging (Ott et al. 1995; Lipnicki et al. 2013), yet older adults differ vastly in their capacity to withstand the aging process (Rapp and Amaral 1992; Norton et al. 2014). Some older adults experience only a gradual drop in cognitive functioning or even no substantial deterioration, while others experience rapid decline and dementia (Hayden et al. 2011). Thus, identifying the neurobiological basis of this heterogeneity would aid to our understanding of optimal neurocognitive aging (Stern 2012) and subsequently help tailor intervention programs to improve brain health and preserve cognitive function in the elderly population, particularly in those who are at increased risk of dementia (Prince et al. 2015).

The term neurocognitive/cognitive reserve, or simply reserve, refers to the observation that older adults who have been exposed to more cognitively stimulating environments are better protected against clinical symptomatology of a variety of neurological conditions, despite substantial disease-related neuropathological changes (Stern et al. 1992; Cabeza et al. 2018; Stern et al. 2018; Xu et al. 2019). In their seminal PET study, Stern et al. (1992) examined regional cerebral blood flow over parietotemporal cortex (as a proxy marker of disease progression) in 3 groups of Alzheimer’s disease patients. The groups were matched on clinical presentation of the disease, but differed according to levels of educational attainment. More advanced AD-related neuropathology (indexed by lower levels of parietotemporal perfusion) was observed in the group with the highest levels of educational attainment, indicating a greater preservation of cognitive function in this cohort, given their relative pathology. The authors proposed that high levels of education resulted in a neuroprotective effect against the clinical symptoms of the disease—a conclusion which has since been substantiated by a large body of epidemiological work highlighting the importance of education to the preservation of neurocognitive function in both healthy and pathological aging conditions (e.g., Ott et al. 1995; Launer et al. 1999; Le Carret et al. 2003; Zhang and Luck 2009). In fact, recently, *The Lancet Commission* for dementia prevention (Livingston et al. 2017) estimated that around the globe as many as 30% of Alzheimer’s disease cases can be linked to 7 risk factors (diabetes, midlife hypertension, midlife obesity, physical inactivity, depression, smoking, and low educational attainment), all of which are modifiable and among which the highest estimated population-attributable risk factor was low educational attainment (Norton et al. 2014). While the original work on cognitive reserve has focused on Alzheimer’s patients (for review see Stern 2012), the term has since been used to account for heterogeneity in neurotypical (healthy) cognitive aging. Moreover, in addition to education, the ongoing research on cognitive reserve in neurotypical aging has highlighted the role of occupational and leisure activities as additional factors offsetting the age-related cognitive decline (i.e., proxies of neurocognitive reserve; for review, see Cabeza et al. 2018).

Despite substantial evidence that exposure to cognitively enriched activities can mitigate the clinical presentation of neuropathological conditions such as Alzheimer’s disease or offset cognitive decline in healthy aging, the neuro-anatomical substrates supporting this phenomenon are unclear. In his pioneering theoretical work on cognitive reserve, Robertson (Robertson 2013, 2014) proposed that a lifetime of engaging noradrenergic-rich cognitive processes strengthens the right-lateralized fronto-parietal networks, which in turn contributes to the behavioral observations of neurocognitive reserve. Cognitively stimulating environments, such as those provided by education, organically necessitate several core cognitive processes including arousal, sustained attention, and response to novelty (Robertson 2014). These cognitive operations rely, to varying extents on the noradrenergic (or locus-coeruleus norepinephrine; LC-NE) system. The LC-NE system shows a strong right lateralization and is functionally linked with the frontoparietal attention networks (Oke et al. 1978; Robinson 1979; Grefkes et al. 2010; Jodo and Aston-Jones 1997; Jodo et al. 1998; Singewald and Philippu 1998; Shalev et al. 2019), particularly the right lateralized “ventral attention network” which encompasses temporoparietal and ventral frontal regions (Corbetta and Shulman 2002; Sara 2009). Computational modeling work using lateralized visual attention tasks in healthy aging (Brosnan, Demaria, et al. 2018), and neuroimaging work with Alzheimer’s patients (van Loenhoud et al. 2017) provided initial support for Robertson’s right hemisphere hypothesis of reserve. However, a unified investigation of cognition, levels of enrichment, and neuroanatomy would provide a comprehensive understanding of the complex interactions between individual differences in cognitive function, environmental influences, and brain structure, an idea which motivated the current study.

In addition to identifying modifiable lifestyle factors that protect against the behavioral manifestations of neuropathology, there has been substantial work investigating how highly specified cognitive capacities decline during the natural aging process (for a comprehensive review see Cabeza et al. 2017). One of the cognitive functions that are strongly affected by aging is attention, as recently reviewed by Zanto and Gazzaley (2017). Age-related declines in attention are heterogeneous and complex, affecting numerous processes and functions such as selective, divided, and sustained attention as well as attentional capture (Zanto and Gazzaley, 2014, 2017). For example, as we age, attentional functions deteriorate resulting in less efficient selection of relevant information and increased distractibility by task irrelevant but salient stimuli. Using the global–local task Tsventanov and colleagues have elegantly demonstrated that, relative to younger adults, older adults have difficulty in attending to task-relevant target attributes in the presence of competing salient distracting information (Tsvetanov et al. 2013). Their findings are in agreement with the inhibitory deficit hypothesis, centered on the idea that aging leads to a selective decrease in inhibitory control, that is, in the ability to block (or inhibit) goal irrelevant information (Hasher et al. 2007; Hasher 2015). This capacity to effectively select and process task-relevant sensory information is fundamental to everyday tasks and deficits in this capacity can cause a cascade of functional issues, as can be observed in Alzheimer’s patients (e.g., Rizzo et al. 2000) and in cases of visual neglect following stroke (e.g., Corbetta and Shulman 2011). Indirect evidence from functional MRI, lesion, and animal neurophysiology work has indicated that the right-lateralized LC-NE system, which has close links with the fronto-parietal network, particularly with the “ventral attention network” (Posner and Petersen 1990; Aston-Jones et al. 1994; Aston-Jones et al. 1997; Corbetta and Shulman 2002; Hurley et al. 2004; Thiebaut de Schotten et al. 2011; Sara and Bouret 2012; Robertson 2014; Chechlacz et al. 2015), critically contributes to the capacity to effectively select visual information from the environment. An unanswered question is whether this right lateralized network, sensitive to proxy measures of neurocognitive reserve, may relate to inter-individual variability in the extent to which some older adults might maintain a youth-like cognitive ability to inhibit salient distracting stimuli as described by Tsvetanov et al. (2013).

In this paper, we ask whether a common right-lateralized anatomical basis underpins cognitive reserve and individual variation in cognitive function (the ability to ignore salient distraction) in those over the age of 65 years. Specifically, we first examine whether reserve, captured by proxies calculated by Cognitive Reserve Index (CRI) questionnaire (Nucci et al. 2012), predicts inter-individual variability in the effect of saliency on attentional selection measured with global–local task (Tsvetanov et al. 2013) in a group of older adults. Subsequently, we employ voxel-based morphometry (Ashburner and Friston 2000; Good et al. 2001) to capture the neural markers of the observed cognitive reserve. We hypothesize that: (1) cognitive reserve would offset age-related deficits in ignoring salient distraction, thereby accounting for heterogeneity in performance in the global–local task and (2) a right lateralized neural substrate of cognitive reserve would underpin individual differences in the capacity to effectively select visual stimuli amid salient distraction.

## Materials and Methods

### Participants

A total of 60 older adults participated in the study (26 males; age range 65–84; mean ± SD age 76.7 ± 4.7), consisting of a magnetic resonance imaging (MRI) session and behavioral testing (the Global Local task and Cognitive Reserve Index questionnaire). All participants were recruited either from the Psychology panel of elderly volunteers or the Birmingham 1000 Elders group, both established at the University of Birmingham. The 2 panels of elderly volunteers consist of adults aged 65 or over who are in good health and have no pre-existing cognitive impairment. All study volunteers had normal or corrected-to-normal vision, had no history of psychiatric or neurological disease, and were right-handed (self-report). Due to MRI contraindications, 10 participants were unable to undergo the MRI scan.

The study was approved by the University of Birmingham Ethical Review Committee. All study participants provided written informed consent and received monetary compensation for participation in agreement with approved ethics protocols.

### Global Local Task

#### Stimuli

**Figure 1:**
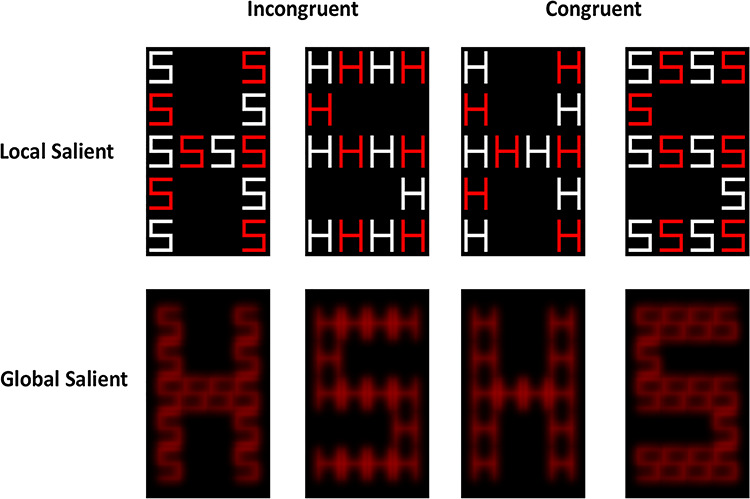
Examples of compound-letter stimuli used in the Global Local task illustrating the congruency (i.e., Global and Local letters either matched or mismatched) and saliency (either high local or high global saliency) manipulations.

We used compound-letter stimuli created by Tsvetanov et al. (2013), based on a previously published global–local task design (Mevorach et al. 2006; Mevorach, Humphreys, et al. 2009; Mevorach, Shalev, et al. 2009; Mevorach et al. 2010). The compound-letter stimuli comprised either the letters “H” or “S” (Local letters) combined into orthogonal letter figure (either “H” or “S” Global letter; see Fig. 1). The compound letters could either be congruent where Local and Global letters matched (i.e., a Global letter “H” comprised Local “H” letters, and a Global letter “S” comprised Local “S” letters) or incongruent where Local and Global letters differed (i.e., either a Global letter “H” comprised “S” letters and *vice versa*; see Fig. 1). The compound letters were then manipulated to enhance the relative saliency of either their “Global” or “Local” attributes. To increase the relative saliency of the “Local” letters, the compound letters were made of red and white letters (Fig. 1 top row; high local saliency). To increase the relative saliency of the “Global” letters, the local letters all appeared in red color and were blurred (Fig. 1 bottom row; high global saliency).

#### Experimental Procedure

**Figure 2:**
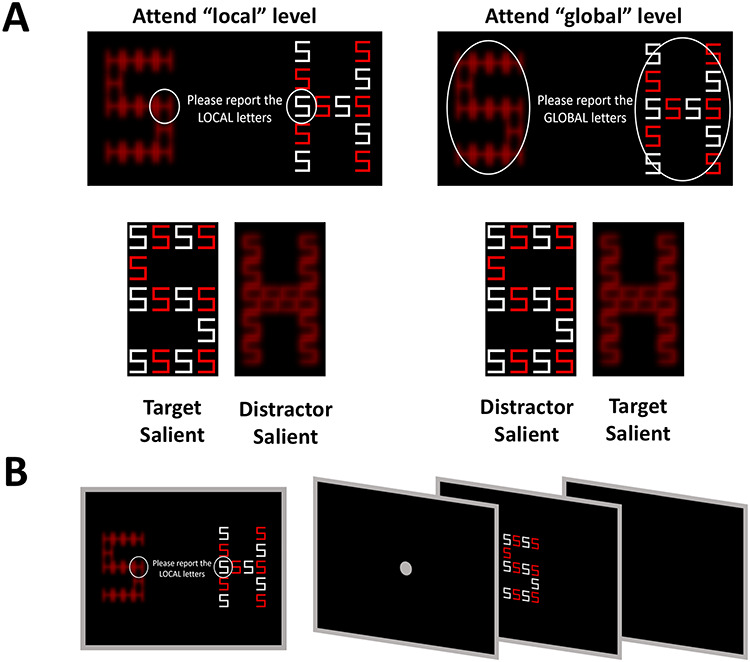
Global Local Task design. (*A*) The experiment consisted of different blocks in which participants were asked to concentrate only on the global or the local level while ignoring the information at the other level. (*B*) Following the instruction screen, on each trial the presentation of a compound-letter was proceeded by a presentation of a fixation point.

At the beginning of each block, participants were instructed to focus either on the global or the local letter’s attribute (as illustrated in Fig. 2*A*), while ignoring the irrelevant information of the other level. They were requested to indicate whether the letter at the attended level (target) was either “H” or “S” by pressing the “H” or “S” key on a computer keyboard, respectively. The unattended stimulus attribute (distractor) was either congruent with respect to the target identity (e.g., both target and distractor were the letter H, or both were the letter S) or incongruent (e.g., target was H and distractor was S, or vice versa). Within each block, 50% of the trials were congruent and 50% were incongruent. In addition, in half of the blocks, the target was salient, whereas in the remaining half of the blocks, the distractor was salient. Overall there were 4 possible block types based on the orthogonal combination of 2 saliency levels (distractor salient or target salient) and the task-relevant stimulus attribute that participants were requested to judge (Global or Local). The experiment commenced with a short demonstration and a practice session of at least 10 trials, or until the experimenter could verify that the instructions were clear and participants were capable of doing the task. During the practice trials, participants were encouraged to ask questions and/or indicate if they had any difficulty, before beginning the experimental run. Each experimental block type was repeated once and had 40 trials. The order of blocks was randomly allocated. Each trial started with a fixation point presented for 1500 ms, and followed by 200 ms of a blank screen. The global–local letter stimulus was then presented for 300 ms and disappeared, leaving a blank screen until the response. The trial ended when participants indicated the letter they identified. The task was administered using Presentation software (Neurobehavioral Systems, Albany, CA). An overview of the task is illustrated in Figure 2*B*.

### Cognitive Reserve

All study participants completed the Cognitive Reserve Index questionnaire (CRIq; Nucci et al. 2012). With this assessment, CR is estimated based on a semi-structured interview aimed at quantifying the amount of CR accumulated throughout the lifetime, based on evaluation of education level as well as day-to-day engagements in professional and leisure activities. The CRIq is a validated measure comprised of 3 subscales assessing education, the complexity of professional activities, and leisure-time activities (Nucci et al. 2012). Based on the frequency and duration (in years) across lifespan of various activities assessed by each subscale, 4 different factors are calculated including overall cognitive reserve index (CRI), CRI education, CRI working activity, and CRI leisure time.

### Statistical Analyses

For the purpose of the statistical analyses, we calculated mean accuracy and response times of correct responses for each individual on each experimental condition (i.e., congruency, target/distractor saliency, and the global/local configuration to attend) of the Global Local Task. As the behavioral performance was measured in adults aged 65 or over, we used accuracy as our main dependent variable to avoid potential motor confounds that are associated with aging (e.g., Roggeveen et al. 2007; Shalev et al. 2016; Haupt et al. 2018). Preliminary data inspection indicated substantial variability in accuracy among experimental conditions as well as inter-individual differences in performance. Thus, the analysis approach based on accuracy measured allowed us to assess attentional capacity without relying on response speed, which is likely to be related to motor difficulties in older age (Roggeveen et al. 2007). Tsvetanov et al. (2013) previously showed age-specific deficits in congruency interference when distractors were salient (Saliency Distraction), irrespective of whether participants were attending global or local attributes of the stimuli. We started our analysis procedure with an ANOVA test to verify the congruency interference in accuracy indices at the group level. This analysis was then followed by a regression analysis, targeting Saliency Distraction as a behavioral marker and assessing its associations with age and cognitive reserve.

#### Validation of the Experimental Manipulation

We carried out a 2 × 2× 2 repeated-measures ANOVA with accuracy rate as the dependent variable, and congruency (congruent vs. incongruent), saliency level (target salient vs. distractor salient), and attended attribute (global/local) as independent factors. This analysis aimed to detect the successful manipulation of global/local congruency, irrespective of saliency manipulation.

#### Assessing the Effect of Age and Cognitive Reserve on Saliency Distraction

We employed a hierarchical regression analysis to address our main research question: whether age and cognitive reserve relate to attentional selection with salient distraction. Based on the ANOVA analysis (as described above), we used the ‘congruency effect’ as a dependant variable in the subsequent regression analysis. Specifically, our dependent variable was the change in accuracy (in percentage) between congruent and incongruent conditions when distractors were salient (Saliency Distraction). This is in line with Tsvetanov et al. (2013) who reported significant age differences between young and older adults particularly for this condition. As predictors, we entered age (in years) as the first block, and then 3 CRI factors (CRI education, CRI working activity, and CRI leisure time) as the second block. The 3 factors were entered in a stepwise approach, to explore the contribution of specific components to the model. This exploratory approach was then supplemented by a Bayesian inference approach, to quantify the strength of the evidence in support of the observed effects (Wetzels et al. 2011; Wagenmakers et al. 2018). Bayesian regression analyses were conducted using an open-source statistical software JASP (Love et al. 2019). When conducting a Bayesian linear regression analysis, we included age in our null model and then added the 3 CRI factors as predictors to observe the potential contribution of each. We reported Bayes factors in favor of the alternative hypothesis, expressing the probability of the data given H1 relative to H0 (BF_10_). We calculated the Bayes Factor Inclusion probabilities (BF_10_) using a Jeffrey–Zellner–Siow prior (using the default prior scale of 0.0354), to verify our exploratory stepwise regression procedure. In addition, we report Bayes factors expressing the likelihood of the specific model against all possible models (BFm). The Bayes factors were subsequently interpreted in accordance with previously published guides (Jeffreys 1961; Zellner and Siow, 1980, 1984; Bayarri and García-Donato 2007). For example, a Bayes factor BF_10_ can be interpreted such that a value of 3 indicates 3 times more support for the alternative hypothesis than the null hypothesis, whereby a value of one-third indicates 3 times more support for the null than the alternative hypothesis (Jeffreys, 1961; Zellner and Siow 1980, 1984; Bayarri and García-Donato 2007).

### MRI Data Acquisition

T1-weighted scans were acquired at the Birmingham University Imaging Centre (BUIC) using a 3-T Philips Achieva MRI system with a 32-channel head coil. T1-weighted structural scans were acquired with a spatial resolution of 1 × 1 × 1 mm^3^ with the following parameters: 176 sagittal slices, TR = 7.5 ms, TE = 3.5 ms and flip angle = 8°.

### MRI Data Pre-Processing and Voxel-Based Morphometry (VBM)

T1-weighted scans from 50 participants (22 males; age range 65–84; mean ± SD age 73.5 ± 4.7) were included in the VBM analyses. The pre-processing of T1 images, as well as all VBM analyses were performed using the FMRIB Software Library (FSL v6.0.1; Smith et al. 2004; Woolrich et al. 2009; Jenkinson et al. 2012). We used an optimized VBM protocol previously used to assessed age-related brain changes (Good et al. 2001) as implemented in the FSL-VBM toolbox (Douaud et al. 2007; http://fsl.fmrib.ox.ac.uk/fsl/fslwiki/FSLVBM). First, the T1 images were brain-extracted to remove non-brain tissue using FSL BET tool (BET v2.1; Smith 2002) and segmented to extract gray matter (GM) prior to registration to MNI 152 standard space using non-linear registration (Andersson et al., 2007). The resulting GM images were averaged and flipped along the x-axis to create a left–right symmetric, study-specific GM template. Subsequently, all individual native GM images were non-linearly registered to this study-specific template and “modulated” to correct for local expansion (or contraction) due to the non-linear component of the spatial transformation. Next, the modulated GM images were smoothed with an isotropic Gaussian kernel with a sigma of 3.5 mm (∼8 mm FWHM). For the purpose of statistical analyses, we used a general linear model (GLM) framework using permutation-based non-parametric testing implemented in FSL-randomized tool (Winkler et al. 2014). All presented findings are based on the whole-brain voxel-wise analyses with recommended 10 000 permutations (Dickie et al. 2015) and with the significance level set at *P* < 0.05 using a threshold-free cluster enhancement method (TFCE; Smith and Nichols 2009) and a family-wise error (FWE) rate correction for multiple comparisons across voxels. We first investigated the effect of age on the regional volumetric GM changes. Further, regression analyses were performed with either Saliency Distraction score, each off the CRI subscales, and a combination of both CRI subscales and the saliency distraction score to explore the GM covariation associated with these measures. In all regression analyses, age and gender were included as covariates and the measures entered into the analysis were demeaned. The cluster tool and the Harvard–Oxford cortical atlas integrated into the FSL image viewer (FSLeyes; https://git.fmrib.ox.ac.uk/fsl/fsleyes/fsleyes/), as well as the Duvernoy Human Brain Atlas (Duvernoy et al. 1991) were used to report results from randomize.

## Results

### Validation of the Experimental Manipulation: Saliency Distraction

Table 1 reports the full descriptive data of the Global Local task including group accuracy and reaction times for all experimental conditions.

**Table 1:** Group performance on the Global Local Task, by each of the ANOVA factors. Means and standard deviations (in parenthesis)

		Accuracy (%)		RT (ms)RT (ms)	
		Congruent	Incongruent	Congruent	Incongruent
Attention to ‘global’	Target Salient	96% (.07)	84% (.27)	735 (238)	809 (244)
	Distractor Salient	98% (.04)	83% (.23)	786 (342)	915 (297)
Attention to ‘local’	Target Salient	98% (.03)	90% (.24)	665 (297)	661 (291)
	Distractor Salient	94% (.13)	87% (.25)	796 (290)	786 (313)

The ANOVA results confirmed the existence of a congruency effect (F(1,59) = 22.400; *P* < 0.001; partial *η*^2^ = 0.275) during the performance of the Global Local task. There were no other main effects or interactions (all *P*’s > 0.09). These findings confirm that accuracy can be used as a marker of cognitive interference in the task. Notably, there was no main effect of the attended attribute factor (global/local; *P* = 0.379) and the attended attribute did not interact with either the Salient *(P* = 0.183) nor Congruency (*P* = 0.143) dimension. Accordingly, in our sample, there was no bias towards either global or local stimulus attributes.

As such, Saliency Distraction (i.e., the change in accuracy between congruent and incongruent conditions when distractors were salient) was use as the dependent variable in subsequent analyses.

### Regression Analysis: The Effect of Age and Cognitive Reserve on Saliency Distraction

A preliminary analysis which included all CRI factors as predictors using a “stepwise” approach led to the inclusion of the CRI education factor and the exclusion of 2 other factors (CRI leisure time *P* = 0.628; CRI working activity *P* = 0.895).

As shown in Table 2, age significantly predicted congruency interference in distractor-salient conditions, that is, Saliency Distraction. This association was positive, meaning that older participants were more likely to experience higher saliency interference. However, when CRI education factor was added as a second predictor, the model fit improved significantly and CRI education appeared to mediate the interference (reflected in a negative slope). These 2 factors did not interact. Interestingly, when estimating the contribution of all Cognitive Reserve factors using a stepwise procedure, we identified a specific contribution of the CRI education, while the 2 other factors were not significant.

**Table 2:** Summary of the final regression model. The stepwise procedure indicated that only the CRI education factor (CRI_E) contributed to the model. The third block included the interaction between CRI-E and Age, and was not significant

	**β**	***R*** ^**2**^	***R*** ^**2**^ **change**	**F change**
**Model #1**		.148	.148	9.363 (*P* = 0.003)
Age	.378^*^			
**Model #2**		.248	.094	6.776 (*P* = 0.012)
Age	.399^*^			
CRI_E	−.307^*^			
**Model #3**		.258	.007	.332 (*P* = 0.482)
Age	1.244			
CRI_E	1.326			
CRI_E X Age	−1.890			

Note: ^*^P < 0.01.

**Figure 3:**
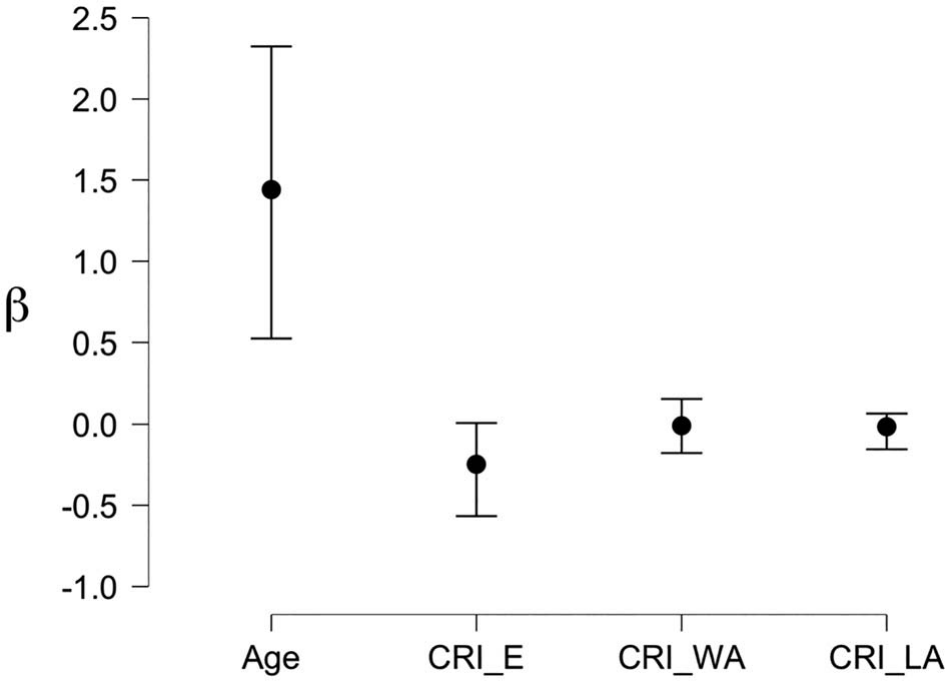
Bayesian regression analysis: Posterior Coefficients with 95% Credible Interval.

We supplemented the above analyses by a Bayesian inference approach, to quantify the strength of the evidence. First, we defined a null-model in which age is the only predictor of congruency interference in high distraction. We then added the 3 CRI factors to compare the models. The results indicated that the CRI education factor had an effect on Saliency Distraction (BF_10_ = 5.869) and contributed to the regression model compared with the null model (BF_M_ = 5.882). All other factors had lower contributions, both independently and combined (all BF_10_ range between 0.359 and 2.091). The posterior coefficients are illustrated in Figure 3.

For completion, we carried out another analysis where we did not define Age as part of the null-model and instead included all factors together and compared with a null model. We found strong evidence in support of a model which includes only the Age and CRI-E factor, when contrasted with the null hypothesis (model *R*^2^ = 0.248; BF_M_ = 11.397; BF_10_ = 77.110).

### VBM Results

**Figure 4:**
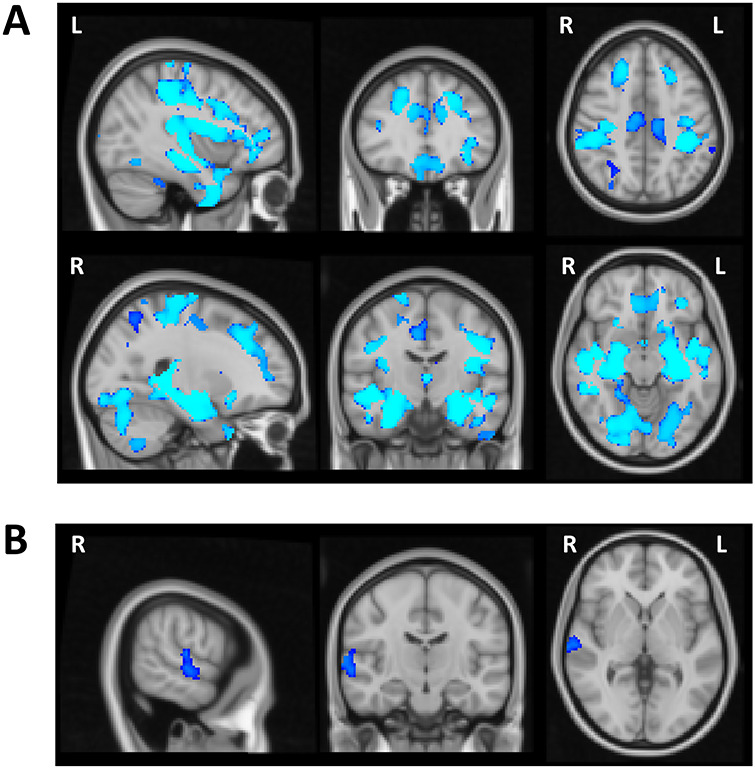
Voxel-wise differences in the gray matter associated with age and cognitive performance in older adults. (*A*) Wide-spread volumetric gray matter differences associated with age (revealing widespread age-related reduction in the gray matter volume). (*B*) Reduced gray matter in the right STG predictive of higher Saliency Distraction in the Global Local task over and above changes in gray matter associated with age.

As anticipated based on prior findings (e.g., Good et al. 2001), VBM analysis showed widespread volumetric GM changes associated with age. There was a significant age-related reduction in the gray matter in several cortical regions within the frontal, parietal, temporal, and occipital lobes bilaterally as well as subcortical regions and the cerebellum (Fig. 4*A*, Table 3). Subsequent regression analysis indicated a relationship between the performance on the Global Local task and GM volume in the right superior temporal gyrus (STG). More specifically, in the examined group of elderly participants reduced GM volume in the right STG was a significant predictor of higher Saliency Distraction (worse performance) on the behavioral task after covarying out the effect of age and gender (Fig. 4*B*, Table 3).

**Table 3:** Local peaks of the significant clusters from the VBM analyses (corrected *P*-value < 0.05) exploring volumetric GM differences associated with age and Saliency Distraction (Global Local Task)

**Model**	**Cortical region**	**MNI coordinates**			***t* value**	**Cluster size (voxels)**
		**X**	**Y**	**Z**		
**Age**						
	Bilateral SFG, MFG, IFG, insula, putamen, STG, MTG, SMG, AG, PC, calcarine, IOG, LG Right Cingulate gyrus Right IPS Left SMG	34 10 24 −60	−10 −14 −64 −40	−36 42 54 46	7.30 3.75 3.37 2.71	32372^*^ 888^*^ 249 17
**Saliency distraction**						
	Right STG	70	−18	4	4.77	276

Note: ^*^Large clusters extending bilaterally*.*

Abbreviations: AG, angular gyrus; IFG, inferior frontal gyrus; IOG, inferior occipital gyrus; IPS, intraparietal sulcus; LG, lingual gyrus; MTG, middle temporal gyrus; PC, precuneus and cuneus; SFG, superior frontal gyrus; SMG, supramarginal gyrus; STG, superior temporal gyrus; VBM, voxel-based morphometry.

**Figure 5:**
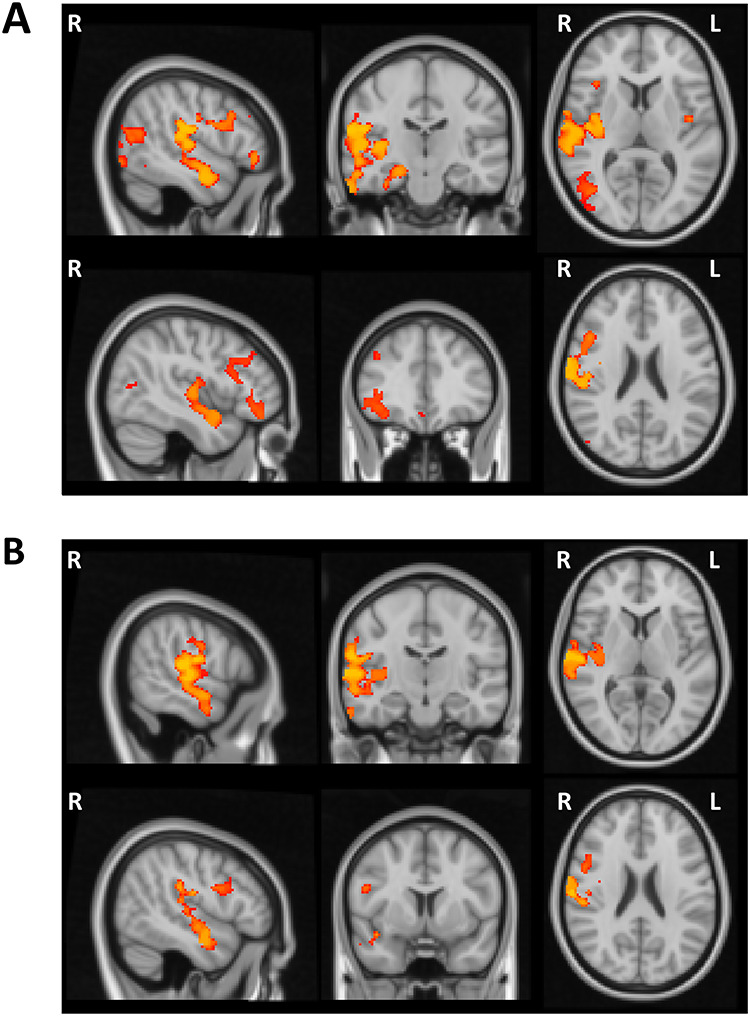
(*A*) Voxel-wise regional difference in gray matter volume as a function of CRI education. (*B*) Regional volumetric gray matter differences associated with CRI education and Saliency Distraction on the Global Local Task. CRI education was associated with greater gray matter volume and better cognitive performance (lower Saliency Distraction).

The analysis of behavioral performance (see above) indicated CRI education factor as a protective factor, that is, longer education was a significant predictor of lower Saliency Distraction in performance on the Global Local Task. The VBM analysis demonstrated a similar protective effect of the CRI education factor on GM volume (i.e., higher CRI education factor was a predictor of higher GM volume), but such an effect was not observed for the other CRI measures (overall CRI, CRI working activity, and CRI leisure time). Specifically, the voxel-wise regression analysis revealed a significant association between higher CRI education and higher GM volume in several cortical regions predominantly within the right hemisphere (Fig. 5*A*). Table 4 reports the cortical regions showing significant associations between GM volume and CRI education factor after correcting for multiple testing, that is, significance level set at *P* < 0.01 based on *Bonferroni* correction. The final regression model including both CRI education and Saliency Distraction showed that lower Saliency Distraction on the Global Local Task in participants with higher CRI education scores was associated with larger GM volume in the right STG (large cluster extending into insular cortex) and temporo-parietal junction (TPJ), as well as right inferior and superior frontal gyri (Fig. 5*B*, Table 4).

**Table 4:** Local peaks of significant clusters from the VBM analyses (corrected *P*-value < 0.01) exploring the effect of CRI education on regional differences in GM volume

**Model**	**Cortical region**	**MNI coordinates**			***t* value**	**Cluster size (voxels)**
		**X**	**Y**	**Z**		
**CRI_E**						
	Right STG, MTG, SMG, insula, OFC, hippocampus Right IFG Left insula Right IPS Right Cingulate gyrus	60 48 −42 48 4	−28 16 −6 −64 34	2 20 18 12 −14	4.89 3.95 5.36 3.91 3.94	4385 439 130 126 35
**CRI_E & saliency distraction**						
	Right STG, insula, TPJ Right IFG Right SFG	68 50 22	−16 8 40	6 22 40	4.97 4.30 4.96	2849 171 83

Note: Abbreviations: CRI_E, cognitive reserve index education factor; IFG, inferior frontal gyrus; MTG, middle temporal gyrus; OFC, orbitofrontal cortex; SFG, superior frontal gyrus; SMG, supramarginal gyrus; STG, superior temporal gyrus; TPJ, temporo-parietal junction; VBM, voxel-based morphometry.

## Discussion

In our study, we combined behavioral (demographic), cognitive, and imaging data to explore predictors of variability in the age-related changes in attentional function, indexed by ability to suppress salient distractions. First, we showed that among older adults (≥65 years old), higher education was associated with reduced interference by salient distractors. We then used imaging data to identify the neural substrates of inter-individual variability in CRI and cognitive measures. Our data revealed right lateralized neural substrates, which underpin the offsetting effects of education (a proxy measure of cognitive reserve) on age-related changes in attention function. The findings provide compelling evidence in support of the hypothesis that a right lateralized network has a key role in cognitive reserve (Robertson 2014).

What has emerged to date from research on aging and attention is the vast complexity and heterogeneous nature of the processes and mechanisms affected. Functional deterioration in different aspects of attention (e.g., selective, sustained, divided) has been linked to different generalized theories of cognitive aging attributing functional decline to slower processing speed, deficits in working memory, and a limitation in perceptual and processing capacities (for review see Zanto and Gazzaley, 2014, 2017). Numerous studies suggest that one important aspect of age-related declines in attention is due to the inefficient selection of relevant information and increased distractibility by irrelevant stimuli (Healey et al., 2008; Schmitz et al., 2010; Campbell et al., 2012; Tsvetanov et al., 2013;). This age-related deterioration in attention is of particular interest as it directly affects the ability to stay focused on current behavioral goals and to inhibit task-irrelevant information, which in turn has a prominent impact on various aspects of daily life. For example, as the ability to suppress irrelevant salient information deteriorates, older adults are more likely to cause accidents when driving or to fall when walking, especially under “multitasking” condition (Gaspar et al. 2013). What also emerges from the literature is a large heterogeneity in the severity of deficits experienced by older adults, as well as conflicting evidence regarding whether specific attention functions are affected by the aging process. Thus, while any insights into factors which could preserve attentional processes and account for the observed heterogeneity of cognitive decline in older population are of pressing interest, a careful selection of tasks examining age-related changes in attention is necessary. Here, we chose to focus on a task measuring age-specific deficits in saliency interference, in accordance with the inhibitory deficit hypothesis. From the viewpoint of the inhibitory deficit hypothesis (inhibitory theory of cognitive aging), aging is associated with a selective decrease in inhibitory control necessary to block goal irrelevant information and suppress unwanted responses (Hasher et al. 2007; Hasher 2015). The loss of inhibitory control in older adults may be compensated for by increased top-down excitatory guidance (Madden et al. 1999; Madden et al. 2004). However, it is unclear to what extent top-down attentional processes are preserved in aging and thus play a compensatory role (Zanto and Gazzaley 2017). Furthermore, orthogonal to any top-down compensatory processes, inhibitory control deficits might still emerge under conditions in which distractors strongly compete for selection with targets, problems which are putatively exacerbated by aging. To account for that, Tsvetanov et al. (2013) used the global–local task to highlight greater interference by salient distractors among a group of older adults, compared with young controls. Subsequently, in our study, we used this saliency-interference effect as a cognitive marker, to examine whether cognitive reserve (CR) predicts inter-individual variability in attention in a group of older participants (≥65 years old).

CR is a theoretical construct used to explain inter-individual differences in susceptibility to cognitive decline associated with disease pathology (e.g., Alzheimer’s disease) or normal aging and as such is not assessed directly but measured by various proxies (Stern et al. 2019). We set out to investigate here whether educational attainment or occupational and/or leisure activities frequently used as proxies of cognitive reserve (Nucci et al. 2012; Stern 2012) could ameliorate the effect of age on saliency distraction in performance of the global–local task in the group of elderly participants. Both age and education (but not other proxy measures of CR) significantly predicted variability in cognitive performance on the global local task, more specifically on the level of congruency interference in the distractor-salient condition. While age was positively associated with interference, meaning that older participants were more likely to experience higher saliency distraction, CRI education was negatively associated with performance, suggesting a neuroprotective role. Thus, our study shows that while interference from salient distractors increases with age, time spent in education accounts for the heterogeneity in the observed age-related deficit in saliency distraction. However, it should be noted that our cross-sectional study design cannot answer the question of whether the protective effect of education relates to the onset and/or rate of decline in the ability to ignore salient distraction. Therefore, future longitudinal studies are needed to determine whether higher education delays decline or prevents an accelerated rate of decline in the capacity to inhibit irrelevant salient information. It is also plausible that education might be associated with initial levels of cognitive ability before old age, which then accounts for the observed heterogeneity in performance later in life. Finally, the ability to be less distractible and to more efficiently select relevant information in early childhood would affect daily functioning and could influence overall scholastic abilities, the time spent in education, thereby indirectly impacting a range of other factors relating to cognitive enrichment across lifespan.

Education is thought to build reserve via strengthening neural resources during childhood and early adulthood, which in turn support maintenance of mental capacities across lifespan (Cabeza et al*.* 2018). By contrast, occupation and leisure activities continue to build up reserve later in life. Our findings, linking variability in saliency interference in attention to education and not professional or leisure activities, indicate that reserve accumulated early in life modulates attention function in older adults and that factors acting at different times during the lifespan may differentially contribute to the maintenance of mental capacities throughout life. However, it should be noted that our findings are limited to one specific aspect of attention measured by performance on the Global Local task. Thus, it is plausible that distinct proxy measures of cognitive reserve might be positively associated with other facets of attention indexed by different cognitive tasks. Moreover, these findings raise a follow up question as to whether distinct cognitive functions (e.g., attention, memory, language) in healthy older adults are homogeneously affected by cognitive reserve in terms of the differential effects of early versus late life factors.

In contrast to other reports indicating a strong relationship between leisure or professional activities and cognitive function in older adults, we did not find similar associations. However, some studies have shown that the relationship between life experiences and cognitive function later in life might differ depending on the type of leisure activities, as well as the broader context of cognitively demanding jobs. Taken together, this perhaps emphasizes the need for more comprehensive evaluation of the effects of various lifestyle and demographic factors on cognition and their interactions, rather than indexing this aspect of cognitive reserve by a single proxy measure (for further discussion, see Foubert-Samier et al. 2012; Clare et al. 2017; Park et al. 2019), which might not accurately reflect the full scope of cognitive enrichment. Overall, measuring cognitive reserve is challenging and up to date several different scale and questionnaire measures have been developed and used, each with different strengths and limitations. A recent systematic review (Kartschmit et al. 2019) of 6 commonly used measures highlighted that somewhat different proxy measures, different lifetime dimensions, and/or different subscales are used to calculate cognitive reserve. The choice of the most appropriate scale or questionnaire is often driven by the specific research question, which unfortunately results in a lack of consistency between studies thereby hindering the comparability of findings (Kartschmit et al. 2019). We opted here for CRIq (Nucci et al. 2012), emphasizing 3 specific dimensions capturing the most commonly used cognitive reserve proxies: education, work activities, and leisure time, measured across entire lifespan, which we used in our previous work (e.g., Brosnan, Demaria, et al. 2018).

To date, few studies have investigated the differential contribution of distinct proxy measures of cognitive reserve on cognitive aging. Interestingly, one such study provides compelling evidence that education but not occupation or leisure activities enhances brain reserve (Foubert-Samier et al. 2012). The authors found a significant relationship between cognitive performance assessed by short verbal fluency test and all 3 cognitive reserve proxy measures. However, in a subsequent VBM analysis they only found group differences in both gray and white matter volume when comparing participants with high relative to low education levels. Foubert-Samier et al. (2012) conclude that early life cognitive stimulation measured by education results in structural brain changes measured by gray and white matter volume, which have measurable effects later in life, that is, constituting a so-called brain reserve.

Our VBM analyses revealed a significant association between education and GM volume, showing larger GM volume in several cortical regions, predominantly within the right hemisphere, in participants with higher CRI education scores. As previously noted, due to the cross-sectional nature of our study, we cannot determine whether the regional volumetric GM differences driven by levels of education result purely from improvements of neural resources in early life or from potentially neuroprotective effects acquired across the whole life span. Similarly to our right-lateralized findings, a recent study in a large group of Alzheimer’s patients found that education correlated with a neuroimaging capture of cognitive reserve modeled as a difference between predicted and observed GM volume and that this effect was most pronounced within the right hemisphere (van Loenhoud et al*.* 2017). Our subsequent analysis demonstrated that lower Saliency Distraction (indicative of better cognitive performance) in participants with higher CRI education scores was associated with higher GM volume in the right STG, right TPJ, and right inferior and superior frontal gyri. These regions are known to be a part of the right-lateralized ventral attention network (Corbetta et al. 2008; Corbetta and Shulman, 2002, 2011), which is tightly linked with the locus-coeruleus noradrenergic system (Jodo and Aston-Jones 1997; Jodo et al*.* 1998; Sara 2009; Sara and Bouret 2012). Our findings indicate that older adults who have been exposed to greater levels of cognitive enrichment, through education, demonstrate increases in gray matter volume within the regions of the right-lateralized ventral attention network, which enhances their capacity to effectively select visual stimuli amid salient distraction. Robertson (2014) proposed that enriched cognitive environments like education necessitate core cognitive processes including arousal and sustained attention which, through their association with the right lateralized noradrenergic system, may strengthen the right fronto-parietal networks resulting in a neuroprotective buffer to cognitive decline. Our results that right-lateralized brain reserve (neural resources strengthen by education) offsets age-related decline ability to ignore distraction provide novel and compelling support for Robertson’s right-hemisphere hypothesis of cognitive reserve. One of the limitations of our study is that we used voxel-based morphometry and thus future work based on measures of either functional or structural connectivity (Fox et al. 2006; Thiebaut de Schotten et al. 2011; Chechlacz et al. 2015; Brosnan et al. 2020) is needed to directly support the notion that the right lateralized fronto-parietal network underpins cognitive reserve.

The concept of brain reserve has been introduced by Stern to highlight inter-individual differences in brain structure as anatomically quantifiable aspects of reserve, distinct from differences in cognitive processes underlying variability in susceptibility to cognitive decline, that is, cognitive reserve (Stern 2009, 2012; Stern et al. 2018; Stern et al. 2019). However, this dichotomous terminoloy predominantly used in dementia research has been objected by some researchers primarily interested in the heterogeneity of cognitive decline in healthy aging. For example, Cabeza and colleagues argue that such dichotomy should be eliminated and replaced by a single-term reserve defined as cumulative cognitive enhancement by genetic, and environmental factors as well as neural resources, which offset age- or disease-related cognitive decline (Cabeza et al. 2018). In the current study we use term “right lateralized brain reserve” purely to describe a lateralized and anatomically quantifiable aspect of reserve, rather than suggesting that the distinction between brain and cognitive reserve should be maintained.

In conclusion, our findings provide novel and important evidence in support of hypothesis that cognitively enriched environments, achieved through education, alter structural organization within right-lateralized fronto-parietal regions, which in turn contributes to the preservation of cognitive function in aging, for example, by offsetting the age-related decline in ability to ignore salient distraction (Robertson, 2014). Our findings add to the increasing body of literature exploring the relative contribution of various sociodemographic and lifestyle factors to cognitive reserve as well as relative contribution of earlier versus later life experiences to cognitive reserve (e.g., James et al. 2011; Foubert-Samier et al. 2012; Clare et al. 2017; Cabeza et al. 2018). Moreover, our study indicates that variability in the capacity to suppress distractors in older adults is driven by the right lateralized neural substrates of brain reserve, encompassing regions within the right frontoparietal attention network (Corbetta and Shulman, 2002). As the ability to inhibit distraction affects many day-to-day cognitive tasks, the right lateralized network could be potentially considered as a marker of neurocognitive health and targeted by neurorehabilitation interventions to enhance daily cognitive functioning in our rapidly growing aging population. While these conclusions are highly speculative, they can be further corroborated by a few recent studies. For example, Moezzi et al. (2019) demonstrated association between cognitive reserve and EEG markers of function connectivity within attention and executive function related networks. Also, crucially, Brosnan et al. (Brosnan, Arvaneh, et al. 2018; Brosnan, Demaria, et al. 2018) showed not only that during tDCS targeting the right frontoparietal network EEG markers of selective attention improved but also that in older adults with lower levels of CR such an intervention significantly alters performance on a visual attention task to resemble that of high reserve individuals.

## Notes

We thank Dr Carmel Mevorach (School of Psychology, University of Birmingham) for the Global Local task materials and for his helpful advice on setting up experimental testing. We also warmly thank the volunteers from the School of Psychology panel and the Birmingham 1000 Elders group for participation in this study. *Conflict of Interest*: None declared.
